# Radiation exposure from CT scans in childhood and subsequent risk of leukaemia and brain tumours: a retrospective cohort study

**DOI:** 10.1016/S0140-6736(12)60815-0

**Published:** 2012-08-04

**Authors:** Mark S Pearce, Jane A Salotti, Mark P Little, Kieran McHugh, Choonsik Lee, Kwang Pyo Kim, Nicola L Howe, Cecile M Ronckers, Preetha Rajaraman, Alan W Craft, Louise Parker, Amy Berrington de González

**Affiliations:** a Institute of Health and Society, Newcastle University, Sir James Spence Institute, Royal Victoria Infirmary, Newcastle upon Tyne, UK; b Northern Institute of Cancer Research, Newcastle University, Sir James Spence Institute, Royal Victoria Infirmary, Newcastle upon Tyne, UK; c Radiation Epidemiology Branch, Division of Cancer Epidemiology and Genetics, National Cancer Institute, Bethesda, MD, USA; d Great Ormond Street Hospital for Children NHS Trust, London, UK; e Department of Nuclear Engineering, Kyung Hee University, Gyeongi-Do, South Korea; f Dutch Childhood Oncology Group—Longterm effects after childhood cancer (DOCG-LATER), The Hague, Netherlands; g Departments of Medicine and Paediatrics, Population Cancer Research Program, Dalhousie University, Halifax, Nova Scotia, Canada

## Abstract

**Background:**

Although CT scans are very useful clinically, potential cancer risks exist from associated ionising radiation, in particular for children who are more radiosensitive than adults. We aimed to assess the excess risk of leukaemia and brain tumours after CT scans in a cohort of children and young adults.

**Methods:**

In our retrospective cohort study, we included patients without previous cancer diagnoses who were first examined with CT in National Health Service (NHS) centres in England, Wales, or Scotland (Great Britain) between 1985 and 2002, when they were younger than 22 years of age. We obtained data for cancer incidence, mortality, and loss to follow-up from the NHS Central Registry from Jan 1, 1985, to Dec 31, 2008. We estimated absorbed brain and red bone marrow doses per CT scan in mGy and assessed excess incidence of leukaemia and brain tumours cancer with Poisson relative risk models. To avoid inclusion of CT scans related to cancer diagnosis, follow-up for leukaemia began 2 years after the first CT and for brain tumours 5 years after the first CT.

**Findings:**

During follow-up, 74 of 178 604 patients were diagnosed with leukaemia and 135 of 176 587 patients were diagnosed with brain tumours. We noted a positive association between radiation dose from CT scans and leukaemia (excess relative risk [ERR] per mGy 0·036, 95% CI 0·005–0·120; p=0·0097) and brain tumours (0·023, 0·010–0·049; p<0·0001). Compared with patients who received a dose of less than 5 mGy, the relative risk of leukaemia for patients who received a cumulative dose of at least 30 mGy (mean dose 51·13 mGy) was 3·18 (95% CI 1·46–6·94) and the relative risk of brain cancer for patients who received a cumulative dose of 50–74 mGy (mean dose 60·42 mGy) was 2·82 (1·33–6·03).

**Interpretation:**

Use of CT scans in children to deliver cumulative doses of about 50 mGy might almost triple the risk of leukaemia and doses of about 60 mGy might triple the risk of brain cancer. Because these cancers are relatively rare, the cumulative absolute risks are small: in the 10 years after the first scan for patients younger than 10 years, one excess case of leukaemia and one excess case of brain tumour per 10 000 head CT scans is estimated to occur. Nevertheless, although clinical benefits should outweigh the small absolute risks, radiation doses from CT scans ought to be kept as low as possible and alternative procedures, which do not involve ionising radiation, should be considered if appropriate.

**Funding:**

US National Cancer Institute and UK Department of Health.

## Introduction

CT imaging is a valuable diagnostic technique, and new clinical applications continue to be identified. As a result, the rates of CT use have increased rapidly in the USA and elsewhere, particularly in the past 10 years.^1^ Although the immediate benefit to the individual patient can be substantial, the relatively high radiation doses associated with CT compared with conventional radiography have raised health concerns.^2^, ^3^, ^4^, ^5^, ^6^, ^7^, ^8^ Potential increases in future cancer risk, attributable to the rapid expansion in CT use have been estimated with risk projection models, which are derived mainly from studies of survivors of the atomic bombs in Japan.^3^, ^6^, ^8^ These studies have been criticised because of concerns about how applicable the findings from this group are to the relatively low doses of radiation exposure from CT scans and to non-Japanese populations. Some investigators claim that there are no risks, or even beneficial effects, associated with low-dose radiation.^9^ No direct studies of cancer risk in patients who have undergone CT scans have been undertaken to date.

We did a study to directly assess the question of whether cancer risks are increased after CT scans in childhood and young adulthood. Here we assess the risks of leukaemia and brain tumours because they are the endpoints of greatest concern as the red bone marrow and brain are highly radiosensitive tissues, especially in childhood.^10^ Furthermore, these tissues are also some of the most highly exposed from childhood CT scans,^11^ and leukaemias and brain tumours are the most common childhood cancers.

## Methods

### Patients and study design

In our observational retrospective cohort study, we included patients without previous malignant disease who were first examined with CT between 1985 and 2002 when they were younger than 22 years of age. Patients were scanned at hospitals within 81 National Health Service (NHS) regional services in Great Britain (England, Wales, and Scotland). We assembled the cohort with historical data from electronic radiology information systems (RIS) from the participating hospitals or, for a small number of patients in five hospitals, from paper or film records. Retrieved data included date of birth, details of the CT examinations, sex, post code, and body parts scanned. We used the patient's identifiers to identify patients having scans in more than one hospital.

This study was approved by the Newcastle and North Tyneside Local Research Ethics Committee (Newcastle upon Tyne, UK) and by the UK National Information Governance Board, exempting the study from requiring individual patient's consent.

### Procedures

Linkage with the NHS Central Registry (NHSCR) provided cancer incidence, mortality and loss-to-follow-up data (eg, notified emigrations) from Jan 1, 1985, to Dec 31, 2008. The NHSCR holds computerised records of everyone registered with an NHS general practitioner in Great Britain (most residents). It is continuously updated with births, deaths, marriages, name changes, and movements of patients, and records cancer incidence from the regional cancer registries. We excluded patients from the cohort who had an exit date of less than 2 years in the case of leukaemia or less than 5 years for brain tumours after the first scan to reduce the possibility of inclusion of patients who had CT scans because a cancer was suspected. We also excluded patients who could not be traced by NHSCR, and those who had missing information or inaccurate information on the date of CT scan.

The appendix shows details of the morphology codes used to define leukaemias. We examined four non-mutually exclusive leukaemia subgroups, which were acute lymphoblastic leukaemia, acute myeloid leukaemia, myelodysplastic syndromes, and leukaemia excluding myelodysplastic syndrome. We defined malignant and benign brain tumours with WHO's International Classification of Diseases for Oncology, 3rd edition topographic codes for meninges, brain, olfactory, and cranial nerves, and other parts of the CNS (spinal tumours were excluded). We examined two subgroups: glioma and meningioma plus schwannoma (appendix).

CT scans deliver very non-uniform radiation doses across the body. Therefore, we assessed the risk of leukaemia and brain tumours in relation to estimated radiation absorbed doses in the appropriate organ (red bone marrow or brain), which were estimated for each type of scan without knowledge of case status. The absorbed dose from a CT scan depends on factors including age, sex, examination type, and year of scan. Data for the machine settings that also influence dose, such as milliampere seconds and peak kilovoltage, were not available for every individual patient from the electronic databases during the study period. Therefore, we obtained typical machine settings for CT in young people from UK-wide surveys undertaken in 1989 and 2003.^11^, ^12^ We combined these data with those from a series of hybrid computational human phantoms^13^ and Monte Carlo radiation transport techniques to estimate absorbed doses to the red bone marrow and brain for reference males and females for integer years of age between 0 and 22 years.^14^, ^15^ Table 1 shows estimated red bone marrow and brain doses from different CT examinations by age and sex after 2001. Dose estimates before 2001 were generally 2–3 times higher than were those after this date because age-specific technical settings were rarely used in earlier years.^12^

**Table 1:** Estimated radiation doses to the brain and red bone marrow from one CT scan, by scan type, sex, and age at scan, as used in this study for scans after 2001

	**Male patients**		**Female patients**	
	Brain dose (mGy)	Red bone marrow dose (mGy)	Brain dose (mGy)	Red bone marrow dose (mGy)
**Age at brain CT**				
0 years	28	8	28	8
5 years	28	9	28	9
10 years	35	6	35	6
15 years	43	4	44	6
20 years	35	2	42	2
**Age at chest CT**				
0 years	0·4	4	0·4	4
5 years	0·3	3	0·3	3
10 years	0·3	3	0·3	3
15 years	0·2	4	0·3	4
20 years	0·2	4	0·3	4
**Age at abdominal CT**				
0 years	0·2	3	0·2	3
5 years	0·1	2	0·1	2
10 years	0·1	3	0·1	3
15 years	0·0	3	0·0	3
20 years	0·0	3	0·0	4
**Age at extremity CT**				
0 years	0·0	1	0·0	1
5 years	0·0	0·2	0·0	0·2
10 years	0·0	0·1	0·0	0·1
15 years	0·0	0·0	0·0	0·0
20 years	0·0	0·0	0·0	0·0

### Statistical analysis

We assessed potential associations between radiation dose and cancer outcomes with Poisson relative risk models fitted by maximum likelihood (see appendix). To avoid inclusion of CT scans related to cancer diagnosis we began accrual of person-time for leukaemia incidence 2 years after the first CT scan and for brain tumours 5 years after the first CT scan. We continued accrual of data until date of first cancer diagnosis or the earliest of death, loss-to-follow-up, or Dec 31, 2008. Because it typically takes at least 2 years for radiation-related leukaemia to develop and 5 years for a solid cancer to develop,^16^ doses were lagged by 2 years for leukaemia and by 5 years for brain tumours. Application of the exclusions and lag periods are described in the appendix. We did sensitivity analyses in which the exclusion and lag periods were increased to 10 years for brain tumours, the follow-up period for leukaemia was decreased from 2008 to 2004, and the age at end of follow-up was restricted to patients younger than 25 years for leukaemia and younger than 28 years for brain tumours. We did significance tests on the basis of the likelihood-ratio test. Unless otherwise stated, we based CIs on the profile likelihood.^17^ When the statistical software failed to produce a convergent profile likelihood bound we used the Wald-based (Fisher information-based) confidence bound. All p values are two-sided and p<0·05 was regarded as significant. We did all statistical analyses with the DATAB and AMFIT modules of the EPICURE programme.^18^

### Role of the funding source

The sponsors of the study had no role in study design, data collection, data analysis, data interpretation, or writing of the report. MSP and ABdG had full access to all the data in the study and had final responsibility for the decision to submit for publication.

## Results

After exclusion of 33 372 patients who could not be traced by NHSCR because of incomplete names or dates of birth in the RIS databases (and 960 non-UK resident patients) and those who were ineligible for follow-up because the exit date occurred less than 2 years in the case of leukaemia analyses or 5 years for brain tumours after the first scan (or when information, such as date of scan, was missing or obviously inaccurate), we included 178 604 individuals in the leukaemia analyses and 176 587 in the brain tumour analyses (table 2).

**Table 2:** Cases of leukaemia and brain tumours and person-years for patients in the assessed cohort

		Leukaemia*		**Brain tumours**†	
		Cases	Person-years	Cases	Person-years
Sex					
	Male	42	953 634	65	657 169
	Female	31	764 937	70	529 372
	Unknown	1	2413	0	1666
Age at first exposure, years					
	0	10	198 052	17	139 414
	1–<5	17	262 437	18	185 942
	5–<10	17	269 369	27	189 415
	10–<15	10	345 320	30	236 891
	≥15	20	645 807	43	436 545
Attained age, years					
	0–<20	47	900 383	65	537 567
	20–<30	23	689 274	53	519 313
	30–<35	2	106 376	12	106 376
	≥35	2	24 951	5	24 951
Years since first exposure					
	0–<10	53	1 266 110	77	733 337
	10–<15	15	347 786	45	347 786
	15–<20	6	101 213	13	101 213
	≥20	0	5871	0	5871
Number of CT scans					
	1	45	1 239 170	72	862 661
	2–4	22	429 324	50	291 192
	≥5	7	52 493	13	34 354
Overall		74	1 720 984	135	1 188 207

Person-year data in the leukaemia group do not sum to the overall number because of rounding.

* Follow-up starting 2 years after first CT scan.

† Follow-up starting 5 years after first CT scan.

We included 283 919 CT scans in the analysis of leukaemia risk, of which 64% (182 337 scans) were of the head. The next most common CT scan types were of the abdomen and/or pelvis (9%, 25 695 scans) and chest CT (7%, 18 910 scans; appendix). The distribution of scan types was very similar for patients in the brain tumour analysis, but the total number of scans was slightly smaller than in the leukaemia analysis because of the longer exclusion period (279 824 scans). Table 2 lists the distributions of cases and overall person-years, by sex, age at first scan, attained age, years since first scan, and the number of scans.

The risk of leukaemia was positively associated with estimated doses delivered by CT scans to the red bone marrow (p=0·0097), as was the risk of brain tumours associated with estimated doses delivered by CT scans to the brain tissue (p<0·0001; figure).

**Figure:**
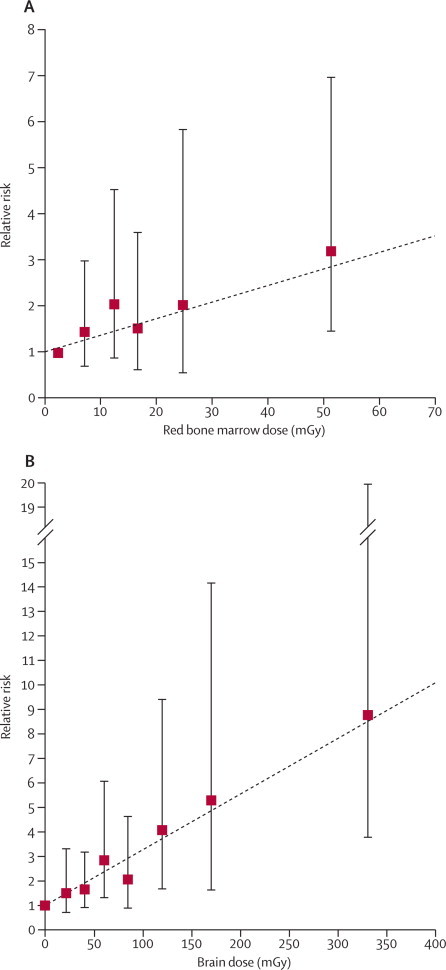
Relative risk of leukaemia and brain tumours in relation to estimated radiation doses to the red bone marrow and brain from CT scans (A) Leukaemia and (B) brain tumours. Dotted line is the fitted linear dose-response model (excess relative risk per mGy). Bars show 95% CIs.

Compared with doses of less than 5 mGy, the relative risk (RR) of leukaemia for patients who received doses of at least 30 mGy (mean dose in this group was 51·13 mGy) was 3·18 (95% CI 1·46–6·94; appendix). Compared with doses of less than 5 mGy, the RR of brain tumours for patients receiving 50–74 mGy (mean dose 60·42 mGy) was 2·82 (1·33–6·03; figure, appendix), and for patients receiving 50 mGy or more (mean dose 104·16 mGy) the brain tumour RR is 3·32 (95% CI 1·84–6·42; appendix). To put this into context, after 2001, 5–10 head CTs in children younger than 15 years result in the accumulation of about 50 mGy red bone marrow dose and 2–3 head CTs results in about a 60 mGy cumulative brain dose (table 1).

We noted positive associations between CT scans and cancer subgroups of gliomas (p=0·0033), schwannoma and meningiomas (p=0·0195), acute lymphoblastic leukaemia (p=0·0053), and myelodysplastic syndromes (p=0·0032), but not acute myeloid leukaemia (p=0·2653) or leukaemia excluding myelodysplastic syndromes (p=0·1436; table 3). For leukaemia, the dose response did not vary between age at exposure, time since exposure, sex, or any other covariates examined (table 4). However, for brain tumours there was significant heterogeneity (p=0·0003) in estimated RR (ERR) across categories of age at exposure, with ERR increasing with increasing age.

**Table 3:** Excess relative risk per mGy for cancer subtypes in relation to organ-specific radiation doses received from CT scans

	**Cases**	**ERR per mGy (95% CI)**	**p value (test for dose-response)**
**Red bone marrow dose**			
All leukaemia, including myelodysplastic syndromes	74	0·036 (0·005 to 0·120)	0·0097
Acute lymphoblastic leukaemia	26	1·719* (>0 to 17·73†)	0·0053
Acute myeloid leukaemia	18	0·021 (–0·042† to 0·155)	0·2653
Myelodysplastic syndromes	9	6·098* (>0 to 145·4†)	0·0032
Leukaemia excluding myelodysplastic syndromes	65	0·019 (–0·012† to 0·079)	0·1436
**Brain dose**			
All brain	135	0·023 (0·010 to 0·049)	<0·0001
Glioma	65	0·019 (0·003 to 0·070)	0·0033
Schwannoma and meningioma	20	0·033 (0·002 to 0·439)	0·0195

ERR=excess relative risk.

* Iteratively reweighted least-squares algorithm failed to converge, so parameter estimates might be unreliable.

† Calculated using Wald-based CI.

**Table 4:** Excess relative risk per mGy for leukaemia and brain tumours, by various personal characteristics

	**Leukaemia**		**Brain tumours**	
	ERR per mGy	p value	ERR per mGy	p value
**Sex**				
Male*	0·031	0·6300	0·016	0·0850
Female	0·042		0·028	
**Years since first exposure**				
0–<5	0·048	0·8061	0†	0·6468
5–<10	0·033		0·025	
≥10	0·026		0·021	
**Years since last exposure**				
0–<5	0·052	0·3004	0†	0·1976
5–<10	0·015		0·026	
≥10	0·014		0·016	
**Number of CT scans**				
1	0·013	0·8013	0·007	0·1213
2–4	0·028		0·021	
≥5	0·035		0·018	
**Age at exposure (years)**‡				
0–<5	0·030	0·5381	0·005	0·0003
5–<10	0·072		0·028	
10–<15	–0·002		0·037	
≥15	0·049		0·041	
**Years since exposure**‡				
2–<5	0·055	0·5357	··	0·2399
5–<10	0·021		0·026	
10–<15	0·005		0·023	
≥15	0·026		0·005	

ERR=excess relative risk. ··=not applicable (follow-up started at 5 years).

* Includes individual of unknown sex.

† Aliased parameter, set to zero.

‡ Time-dependent variable.

We noted little evidence of non-linearity of the dose-response, using either linear-quadratic or linear-exponential forms of departure from linearity (leukaemia exponential p=0·2672 and quadratic p=0·4683, brain tumour exponential p=0·9203 and quadratic p=0·8993). In sensitivity analyses in which all scans 10 years before brain tumour diagnosis were excluded, the magnitude of the dose-responses was increased rather than decreased as might be expected if the association was driven by bias from CT scans related to the diagnosis (appendix). When follow-up for leukaemia was restricted to 2004, the dose-response also increased, which was as expected given the short latency period for leukaemia and early peak in excess risk reported in previous studies.^10^, ^16^ To assess whether the missing exposure data after age 22 years resulted in underestimation of doses and hence overestimation of the relative risks, we restricted follow-up to individuals younger than 28 years for brain tumours and individuals younger than 25 years for leukaemia, but this did not change the dose-response estimates.

## Discussion

In this retrospective cohort study, we show significant associations between the estimated radiation doses provided by CT scans to red bone marrow and brain and subsequent incidence of leukaemia and brain tumours. Assuming typical doses for scans done after 2001 in children aged younger than 15 years, cumulative ionising radiation doses from 2–3 head CTs (ie, ∼60 mGy) could almost triple the risk of brain tumours and 5–10 head CTs (∼50 mGy) could triple the risk of leukaemia.

Although no previous cohort studies have assessed the risk of cancer after CT, several studies have reported significantly increased cancer risks after radiation exposure in the range received from multiple CT scans (100 mGy).^19^ Such studies include those of survivors of the atomic bombs in Japan,^20^ nuclear workers,^21^ and patients who received tens of diagnostic radiographs.^22^ A few case-control studies have also assessed cancer risks from CT scans on the basis of self-reported history of diagnostic radiograph exposures.^23^, ^24^ These studies might be subject to recall bias whereby patients are more likely to recall previous medical radiation exposures than are unaffected controls, and also high levels of reporting error. We avoided such bias by taking a cohort approach and assessing more accurate exposure histories from medical records (panel).

Panel **Research in context**

**Systematic review**

We searched PubMed and Medline databases without date or language restriction for articles with the search terms “computed tomography”, “ionizing radiation”, “cancer”, “radiation-induced neoplasms”, “case-control”, and “prospective”. We reviewed reports from scientific committees such as the International Commission on Radiological Protection (ICRP), United Nations Scientific Committee on the Effects of Atomic Radiation (UNSCEAR), and Biological Effects of Ionizing Radiations (BEIR), and also a broader range of publications and reports covering medical imaging and radiation exposure. We checked references from selected publications for relevance to this study including comments, correspondence, and editorials. Exposure to ionising radiation is an established risk factor for leukaemia and brain tumours.^10^, ^16^ Although CT has important clinical uses, concerns exist about the potential cancer risks from the associated ionising radiation, particularly for children. Rates of CT use have been rising rapidly in the developed world.

**Interpretation**

Increases that we noted in incidence rates of leukaemia and brain tumours after childhood exposure to CT scans are unlikely to be due to confounding factors. The evaluated risks per unit dose were consistent with those derived from recent analyses of cohorts exposed to higher average radiation doses and dose rates. The current study supports the extrapolation of such risk models to doses from CT scans.

In terms of the quantitative estimates of the risk, our primary comparison for leukaemias and brain tumours is with the Life Span Study^20^ of Japanese atomic bomb survivors, which is the most comprehensive study of cancer after radiation exposure currently available.^10^, ^16^ The dose-response for leukaemia following childhood exposure and similar follow-up time (<15 years after exposure) in the Life Span Study was 0·045 per mSv (95% CI 0·016–0·188; appendix) which was much the same as our estimate (ERR of 0·036 per mGy [0·005–0·120]; 1 mSv=1 mGy). For brain tumours, our result (ERR 0·023 per mGy [0·010–0·049]) was about four times higher than was the Life Span Study estimate (0·0061 per mSv [0·0001–0·0639] <20 years after exposure; appendix), but the CIs are wide and overlapped. We had reduced power to examine risks by subtype of neoplasm, age, or time since exposure compared with the Life Span Study, partly because of the more restricted ranges of length of follow-up and age at exposure. The increased risks noted in our study compared with the Life Span Study might be because existing tumours in some patients were not detected at the time of their first CT. The relatively low-energy x-radiation from CT scans might also be about twice as biologically effective per unit dose as the mainly high-energy γ-rays that were the predominant exposure source from the atomic bombings in Hiroshima and Nagasaki.^16^

Our large study sample was collected from a wide range of hospitals in Great Britain. Because most medical attendances at hospitals in Great Britain, particularly for the age group in this study, are in public, free-to-access, NHS hospitals, the sample is probably representative of the childhood and young adult population in the country as a whole who undergo CT. Ascertainment of cancer diagnoses by NHSCR is estimated to be 97%^25^ and therefore there is a low likelihood of losses to follow-up. Patients who were excluded because linkage to their records was not possible had similar characteristics to those who were linked and thus should not have biased conclusions. Because we assessed children and young adults, our results are directly applicable to a highly radiosensitive section of the population,^10^ although whether the results can be generalised to adulthood CT scans has not been established. Moreover, because most (>80%) of the population assessed was white, whether the results are generalisable to other ethnic groups is unknown.

CT is often used as a diagnostic technique when a solid cancer is suspected. However, information about the reasons for CTs and other clinical variables were not available for this study. Instead, we excluded all scans undertaken in the 2 years before a leukaemia diagnosis and 5 years before a brain tumour diagnosis. Young patients with leukaemia are unlikely to have a CT because of their disease,^26^ but we still used a cautious approach of applying an exclusion period. By contrast, patients with brain tumours will probably have a number of CT examinations during the diagnostic period, hence the longer exclusion period. Nevertheless, we noted much the same results in sensitivity analyses in which all scans in the 10 years before a brain tumour diagnosis were excluded. The absence of data for other exposures, such as radiographs, is unlikely to have introduced a major bias because the doses from these scans are typically ten-times smaller than those for CT scans. However, we cannot rule out this bias and the increased dose response noted for brain tumours compared with the survivors of the atomic bombs in Japan is also a possible indication of some residual bias despite the long exclusion period.

Previous dose estimates for CT typically provided effective dose rather than organ doses and were restricted in terms of the ages covered. In this study, a series of phantoms with a higher age resolution from newborn to adult was used for both males and females. We also used more realistic anatomy and bone marrow dosimetry models compared with previous computational phantoms. These advanced features allow more accurate and valid estimates of organ-specific doses. Despite these advanced methods, uncertainties exist for our dose estimates. However, such uncertainties are likely to be mainly Berksonian (resulting from applying group-averaged estimates), and thus would not be expected to bias the dose response.^27^ Collection of detailed scan parameter data for individual patients was not possible. Instead, we used average CT machine settings from two national surveys and assumed that no technical adjustment was made for paediatric patients before 2001.^5^

Absolute excess risk estimates are necessary to put the risks into perspective with the benefits of the scans. Good evidence from the long-term study of the atomic bomb survivors in Japan suggests that cancer risk persists indefinitely after radiation exposure and most cancer types are inducible by radiation.^10^, ^16^ At present, we only have sufficient case numbers to assess brain tumours and leukaemia, and the maximum age of patients at the end of follow-up is 45 years, with a minimum age of 6 years and maximum follow-up time of 23 years. Provisional estimates of excess absolute risk for the end of follow-up at about 10 years after exposure suggest that, of 10 000 people between the ages of 0–20 years receiving 10 mGy from a CT scan, there would be about 0·83 (95% CI 0·12–2·77) excess leukaemia cases and 0·32 (0·14–0·69) excess brain tumours (appendix). Applying the dose estimates for one head CT scan before the age of 10 years (table 1) this estimate would translate into approximately one excess case of leukaemia and one excess brain tumour per 10 000 patients. Increased follow-up and analysis of other cancer types is needed to identify the lifetime excess cancer risk associated with CT scans. Some evidence^28^ suggests that doses in the range delivered by several CT scans might increase the risk of cardiovascular disease. Investigating this feature would require not only the same long-term follow-up required for adulthood cancer outcomes, but also a new approach to obtain cardiovascular incidence data, which is not currently recorded on a registry rather than reliance on mortality data.

Various studies have estimated the potential lifetime excess cancer risks from CT scans from risk projection models, which are largely based on risk models from studies of survivors of the atomic bombs in Japan. Because our relative risk estimates are broadly consistent with the results from the Life Span Study, this study provides additional direct support for the existing lifetime absolute cancer risk projections for paediatric patients.^3^, ^7^, ^8^, ^29^ The most recent risk projections^8^ suggest that, for children with normal life expectancy, the lifetime excess risk of any incident cancer for a head CT scan (with typical dose levels used in the USA) is about one cancer per 1000 head CT scans for young children (<5 years), decreasing to about one cancer per 2000 scans for exposure at age 15 years. For an abdominal or pelvic CT scan, the lifetime risks for children are one cancer per 500 scans irrespective of age at exposure. These absolute excess lifetime cancer risks (to age 100 years) are very small compared with the lifetime risk of developing cancer in the general population, which is about one in three, and are also likely to be small compared with the benefits of the scan, providing it is clinically justified.^1^

We estimated doses for each scan that every patient received, obtained outcome data for the patients, and provided direct evidence that doses at the level children and young adults can receive from CT are associated with increased risks of leukaemia and brain tumours. The dose-response relation that we noted and relative risks of more than 2 for an exposure that is an established carcinogen at higher dose-levels^10^, ^16^ is evidence that this relation is unlikely to be entirely due to confounding factors. With the increasing use of CT worldwide, particularly within this young population,^8^ knowledge of the risks based on empirical data will be crucial to assess safety in relation to the benefits that CT provides. Frequent calls have been made to decrease doses, following the as low as reasonably achievable (ALARA) principle, and only scan when justified as in the current image gently campaign.^30^ In the UK, the Ionising Radiation (Medical Exposure) Regulations mean that a CT scan should only be done when clinically justified, which might explain the low levels of CT use in the UK compared with other countries that do not have such regulations. The immediate benefits of CT outweigh the long-term risks in many settings^31^ and because of CT's diagnostic accuracy and speed of scanning, notably removing the need for anaesthesia and sedation in young patients, it will remain in widespread practice for the foreseeable future. Further refinements to allow reduction in CT doses should be a priority, not only for the radiology community but also for manufacturers. Alternative diagnostic procedures that do not involve ionising radiation exposure, such as ultrasound and MRI might be appropriate in some clinical settings.
