# Supplementary material for: Radiation exposure from CT scans in childhood and subsequent risk of leukaemia and brain tumours: a retrospective cohort study
Source: Lancet. 2012 Aug 4;380(9840):499–505. doi: 10.1016/S0140-6736(12)60815-0 (PMC3418594; doi:10.1016/S0140-6736(12)60815-0)
Supplement: Supplementary appendix [file mmc1.pdf]

THE LANCET

Supplementary appendix

This appendix formed part of the original submission and has been peer reviewed. We post it as supplied by the authors.

Supplement to: Pearce MS, Salotti JA, Little MP, et al. Radiation exposure from CT scans in childhood and subsequent risk of leukaemia and brain tumours: a retrospective cohort study. *Lancet* 2012; published online June 7. DOI:10.1016/S0140-6736(12)60815-0.

Appendix 1. Leukaemia morphology codes (all behaviour code 3 unless otherwise stated)

Analysis sub-groups[a]	Leukaemia sub-group	Morphology
b,d	acute myeloid leukaemia	9840, 9861, 9866, 9867, 9870-9874, 9891, 9895-9897, 9910, 9920, 9930, 9931
c	myelodysplastic syndromes	9980, 9982-9987, 9989
d	chronic myeloid leukaemia	9863, 9875, 9876
d	other myeloproliferative disorders	9950, 9960-9964, 9975·1
a,d	acute lymphoblastic leukaemia/precursor B-cell lymphoblastic leukaemia	9727-9729, 9835-9837
d	myeloid (not otherwise specified (NOS)	9860
d	chronic myelomonocytic leukaemia, NOS	9945
d	juvenile myelomonocytic leukaemia	9946
d	lymphoid, NOS	9820
d	mixed myeloid/lymphoid lineage	9805
d	acute leukaemia, NOS	9801
d	leukaemia, NOS	9800

[a] a) acute lymphoblastic leukaemia, b) acute myeloid leukaemia, (c) myelodysplastic syndromes, and (d) leukaemia excluding myelodysplastic syndrome

Appendix 2. Brain tumour sub-group topography and morphology codes

Analysis sub-groups[a]	Brain sub-group	Morphology
a	glioma	9380-9480
b	meningioma	9530-9539
b	schwannoma	9560.0
	other brain	All brain and central nervous system morphology codes excluding those above

[a]Two analysis sub-groups were used a) Glioma and b) Meningioma plus schwannoma.

The following topography codes were used: C70.0, C70.9, C71.0-C71.9, C72.2-C72.5, C72.8, C72.9 for all sub-groups except meningioma which only included C70.0 & C70.9 and any behaviour code unless otherwise stated.

Appendix 3. More detailed statistical methods.

A Poisson relative risk model was fitted by Poisson maximum likelihood (McCullagh & Nelder, 1989) in which the expected number of cases in stratum i with average attained age, a_i, dose $(D_{i,j})_{j=1}^{4}$ (in Gy) accumulated in four age-at-exposure groups ($j=1=$ age 0-4 years at exposure, $j=2=$ age 5-9 years at exposure, $j=3=$ age 10-14 years at exposure, $j=4=$ age 15+ years at exposure) is:

$$PY_i \exp\left[\sum_{i=1}^{8}\chi_i \ln(a_i / 21)^i + \sum_{j=1}^{N}\eta_j Z_{ji}\right]\left[1+\sum_{j=1}^{4}(\alpha D_{i,j} + \beta D_{i,j}{}^2)\exp\left(\sum_{j=1}^{N}\varphi_j Z_{ji}\right)\right] \quad (1)$$

For other covariates, $\left(Z_{ji}\right)$, i.e., sex, SES quintile, time since first exposure. PY_i is the number of person years of follow-up in that stratum. Attained age, at time of exiting the study through a cancer diagnosis, death or loss-to-follow-up, or at the end of the study period, was centered by dividing by its approximate mean value, 21 years in order to stabilise regression parameter estimates. A similar model was fitted to allow for possible differing contributions by time before the cancer diagnosis, in which the $(D_{i,j})_{j=1}^{4}$ (in Gy) represented the dose accumulated in four periods prior to cancer diagnosis: $j=1=$ 2-4 years, $j=2=$ 5-9 years, $j=3=$ 10-14 years, and $j=4=$ more than 15 years before diagnosis).

A variant model was fitted to leukaemia data, in which:

$$PY_i \exp\left[\begin{array}{l}\chi_1 1_{0\le a_i<5} + \chi_2 1_{5\le a_i<10} + \chi_3 1_{10\le a_i<20} \\ +\chi_4 1_{20\le a_i<30} + \chi_5 1_{30\le a_i} + \sum_{j=1}^{N}\eta_j Z_{ji}\end{array}\right]\left[1+\sum_{j=1}^{4}(\alpha D_{i,j} + \beta D_{i,j}{}^2)\exp\left(\sum_{j=1}^{N}\varphi_j Z_{ji}\right)\right] \quad (2)$$

Reference

McCullagh P, Nelder JA. Generalized linear models. 2nd edition. Monographs on statistics and applied probability 37. 1-526. 1989. Boca Raton, FA, Chapman and Hall/CRC.

Appendix 4. Examples of exclusions and lag periods for follow-up for brain tumours with an exclusion period of 5 years after 1st CT scan and a lag period of 5 years for each CT scan

a) Patient excluded because they died or developed cancer within 5 yrs of 1st CT

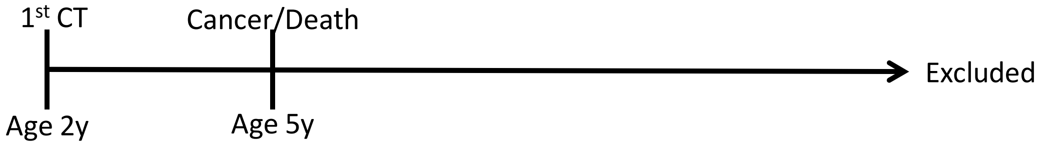

b) Patient included, follow-up starts at age 7 and cancer /death at age 11

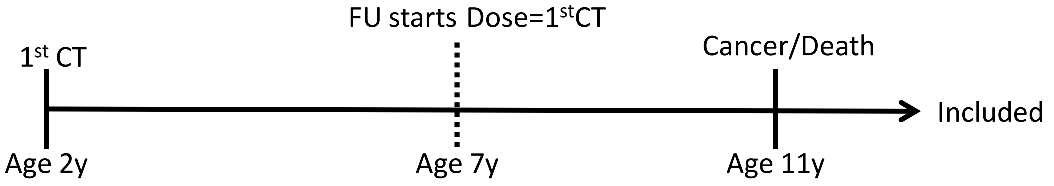

c) Patient included, follow-up starts age 7 for 1st CT and cancer /death age 11, 2nd CT dose not included

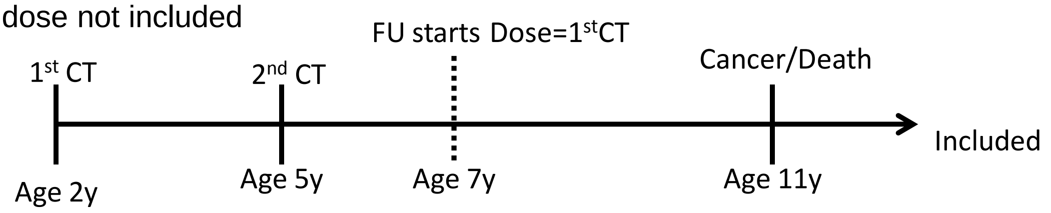

d) Patient included, follow-up starts age 7 for 1st CT and age12 for 2nd CT

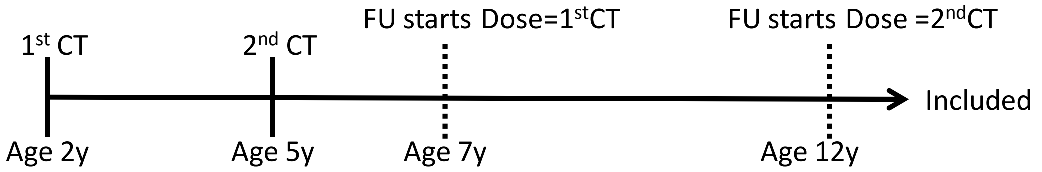

e) Patient excluded as they had cancer before the 1st CT

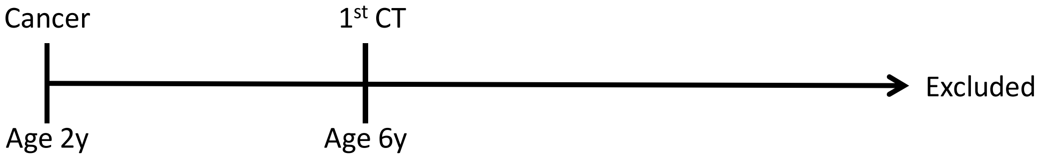

Appendix 5. Types of CT scans that the patients underwent, in the leukaemia analysis (a) and the brain tumours analysis (b)*, by age at CT scan

a) Leukaemia

	Age 0<5		Age 5-<9		Age 10-<15		Age 15-21		Total	
CT scan type	n	%	n	%	n	%	n	%	n	%
Head	50179	66.0%	25794	58.7%	30341	53.5%	55621	51.9%	161935	57.0%
Partial head	3067	4.0%	3632	8.3%	5286	9.3%	8417	7.8%	20402	7.2%
Facial bone	1108	1.5%	726	1.7%	1071	1.9%	2319	2.2%	5224	1.8%
Abdomen	2404	3.2%	1914	4.4%	2712	4.8%	7960	7.4%	14990	5.3%
Abdomen + pelvis	211	0.3%	235	0.5%	475	0.8%	1435	1.3%	2356	0.8%
Pelvis	1100	1.4%	660	1.5%	1360	2.4%	5229	4.9%	8349	2.9%
Chest	4658	6.1%	2676	6.1%	3062	5.4%	6048	5.6%	16444	5.8%
HRCT	772	1.0%	685	1.6%	476	0.8%	533	0.5%	2466	0.9%
Whole body	107	0.1%	75	0.2%	96	0.2%	536	0.5%	814	0.3%
Cervical spine	848	1.1%	687	1.6%	1061	1.9%	2330	2.2%	4926	1.7%
Lumbar spine	74	0.1%	79	0.2%	314	0.6%	1414	1.3%	1881	0.7%
Thoracic spine	21	0.0%	17	0.0%	49	0.1%	169	0.2%	256	0.1%
Spine	512	0.7%	592	1.3%	1167	2.1%	3335	3.1%	5606	2.0%
Extremity	2665	3.5%	2477	5.6%	5561	9.8%	7418	6.9%	18121	6.4%
Hip	786	1.0%	158	0.4%	289	0.5%	402	0.4%	1635	0.6%
Shoulder	19	0.0%	43	0.1%	119	0.2%	281	0.3%	462	0.2%
Total	75982	100.0%	43937	100.0%	56753	100.0%	107247	100.0%	283919	100.0%

b) Brain tumours

	Age 0<5		Age 5-<9		Age 10-<15		Age 15-21		Total	
CT scan type	n	%	n	%	n	%	n	%	n	%
Head	49333	66.1%	25526	58.8%	30088	53.5%	54776	51.9%	159723	57.1%
Partial head	3034	4.1%	3609	8.3%	5250	9.3%	8335	7.9%	20228	7.2%
Facial bone	1101	1.5%	724	1.7%	1068	1.9%	2292	2.2%	5185	1.9%
Abdomen	2329	3.1%	1875	4.3%	2679	4.8%	7786	7.4%	14669	5.2%
Abdomen + pelvis	1081	1.4%	646	1.5%	1347	2.4%	5154	4.9%	8228	2.9%
Pelvis	211	0.3%	232	0.5%	472	0.8%	1399	1.3%	2314	0.8%
Chest	4573	6.1%	2616	6.0%	2977	5.3%	5874	5.6%	16040	5.7%
HRCT	767	1.0%	680	1.6%	475	0.8%	514	0.5%	2436	0.9%
Whole body	106	0.1%	71	0.2%	92	0.2%	515	0.5%	784	0.3%
Cervical spine	832	1.1%	682	1.6%	1055	1.9%	2297	2.2%	4866	1.7%
Lumbar spine	74	0.1%	79	0.2%	314	0.6%	1404	1.3%	1871	0.7%
Thoracic spine	21	0.0%	17	0.0%	49	0.1%	165	0.2%	252	0.1%
Spine	503	0.7%	589	1.4%	1163	2.1%	3292	3.1%	5547	2.0%
Extremity	2651	3.6%	2455	5.7%	5526	9.8%	7338	7.0%	17970	6.4%
Hip	782	1.0%	158	0.4%	289	0.5%	400	0.4%	1629	0.6%
Shoulder	19	0.0%	43	0.1%	119	0.2%	278	0.3%	459	0.2%
Total	74658	100.0%	43427	100.0%	56205	100.0%	105534	100.0%	279824	100.0%

*The number of CT scans is smaller in the brain tumour analysis because the exclusion and lag period was longer (5 years rather than 2 years in the leukaemia analysis).

Appendix 6. Relative risk (and 95% CIs) for leukaemia and brain tumours in relation to red bone marrow and brain categories dose, respectively

a) Leukaemia dose response

	Dose group (mGy)					
	<5	5-9	10-14	15-19	20-29	30+
Cases	15	17	12	11	4	15
Person-years	588450	438828	213289	244844	70523	165049
Mean dose per group (mGy)	2·32	7·08	12·34	16·54	24·69	51·13
Relative risk (95% CI)	1	1·44 (0·70, 2·99)	2·03 (0·89, 4·54)	1·53 (0·63, 3·59)	2·02 (0·56, 5·83)	3·18 (1·46, 6·94)

b) Brain cancer dose response

	Dose group (mGy)							
	<5	5-29	30-49	50-74	75-99	100-149	150-199	200+
Cases	13	14	55	16	11	10	5	11
Person-years	219936	157591	545416	100203	88554	41209	15538	19760
Mean dose per group (mGy)	0·25	21·38	40·29	60·42	84·36	119·77	170·22	330·18
Relative risk (95% CI)	1	1.53 (0·72, 3·30)	1.65 (0·93, 3·16)	2.82 (1·34, 6·03)	2.05 (0·90, 4·62)	4.05 (1·71, 9·33)	5.26 (1·68,14·11)	8.74 (3·77, 19·91)

c) Brain cancer dose response (collapsed upper dose group)

	Dose group (mGy)			
	<5	5-29	30-49	50+
Cases	13	14	55	53
Person-years	219936	157591	545416	265264
Mean dose per group (mGy)	0·25	21·38	40·29	104·16
Relative risk (95% CI)	1	1.53 (0·72, 3·30)	1.64 (0·92, 3·14)	3.32 (1·84, 6·42)

Appendix 7. Sensitivity analyses: Excess relative risks (ERR) for cancer subtypes in relation to organ-specific radiation doses received from CT scans by young people in Great Britain, 1985 to 2002.

	Cases	ERR (/mGy red bone marrow dose) (95% CI)	p-value (test for dose)
**Leukaemia follow-up to 31/12/2004, 2 year lag, 2 year exclusions**			
All leukaemias including myelodysplastic syndromes	48	0·1073 (0·0228, 0·6255)	0·0003
Acute lymphoblastic leukaemia	22	1·5020[a] (>0, 15·66[b])	0·0029
Acute myeloid leukaemia	10	0·2588 (0·0100, 8·001)	0·0150
Myelodysplastic syndromes	7	4·486[a] (>0, 96·70[b])	0·0048
All leukaemias excluding myelodysplastic syndromes	41	0·0660 (0·0077, 0·3802)	0·0101
**Leukaemia follow-up to 31/12/2008, 2 year lag, 2 year exclusions, age < 25**			
All leukaemias including myelodysplastic syndromes	60	0·0539 (0·0097, 0·2243)	0·0033
**Leukaemia follow-up to 31/12/2008, 2 year lag, 2 year exclusions, no scans in 2002**			
All leukaemias including myelodysplastic syndromes	68	0·0231 (-0·0005, 0·0869)	0·0594
**Brain cancer follow-up to 31/12/2008, 5 year lag, 10 year exclusions**			
	Cases	ERR (/mGy brain dose) (95% CI)	p-value (test for dose)
All brain	59	0·0447 (0·0119, 0·2107)	<0·0001
Glioma	27	0·0119 (-0·0119[b], 0·0850)	0·1143
Schwannoma + meningioma (+meningioma NOS)	12	0·0442 (-0·0764[b], 19·22)	0·0963
**Brain cancer follow-up to 31/12/2008, 5 year lag, 5 year exclusions, age < 28**			
All brain	104	0·0194 (0·0074, 0·0466)	<0·0001
**Brain cancer follow-up to 31/12/2008, 5 year lag, 5 year exclusions, no scans in 2002**			
All brain	134	0·0245 (0·0106, 0·0535)	<0·0001

[a]indications that the iteratively reweighted least-squares algorithm failed to converge, so that parameter estimates may be unreliable
[b]Wald-based CI

Appendix 8. Excess risk in the Japanese atomic bomb survivor Life Span Study (LSS) incidence data.

The publicly downloadable data relating to the latest published analysis of the leukaemia (Preston et al, 1994) and solid cancer (Preston et al, 2007) LSS incidence data was used.

A linear relative risk model was fitted to both datasets, with semi-parametric background model adjusted for city, sex, age at exposure and time since exposure (for leukaemia) and for city, sex, age at exposure and age (for brain). In both cases, and as with many recent RERF analyses, the so-called not-in-city group of survivors were excluded, as these are thought to be socioeconomically distinct from the exposed group, as also those with kerma doses > 4 Gy, which are thought to be less reliable. The appropriate organ dose was used, and in each case a neutron relative biological effectiveness of 10 was used.

Excess relative risk /mSv (and 95% CI) in fits to leukaemia and brain cancer

**Leukaemia (AML+CML+ALL)**	ERR (/mSv red bone marrow dose) (95% CI)
Age at exposure < 20, all follow-up	0·01035 (0·00538, 0·01997)
Age at exposure < 20, follow-up < 20 years after exposure	0·03708 (0·01422, 0·1272)
Age at exposure < 20, follow-up < 15 years after exposure	0·04505 (0·01573, 0·1882)
**Brain cancer**	ERR (/mSv brain dose) (95% CI)
Age at exposure < 20, all follow-up	0·00095 (0·00017, 0·00219)
Age at exposure < 20, follow-up < 20 years after exposure	0·00614 (0·00012, 0·06393)

References

Preston DL, Kusumi S, Tomonaga M et al. Cancer incidence in atomic bomb survivors. Part III. Leukemia, lymphoma and multiple myeloma, 1950-1987. Radiat Res 1994;137:S68-S97.

Preston DL, Ron E, Tokuoka S et al. Solid cancer incidence in atomic bomb survivors: 1958-1998. Radiat Res 2007;168:1-64

Appendix 9. Population risks for UK population (2001 incidence rates, 2003 mortality rates) predicted by nominal leukaemia and brain/CNS relative risk coefficients

Risks (% radiation exposure induced cancer incidence (REIC)/ radiation exposure induced death (REID)) to UK population (2001 incidence, 2003 mortality) (and 95% CI) by period of expression (after exposure) from an organ dose of 10 mGy given at age 0-19, assuming nominal risk coefficients of leukaemia and brain/CNS cancer derived from the present study. These risks can be combined with the dose estimates per scan from Table 1 to estimate cumulative absolute risk estimates for specific exposure scenarios.

Expression period (years after exposure)	Incidence	Mortality
	Leukaemia	
2-10	0.0083 (0.0012, 0.0277)	0.0030 (0.0004, 0.0099)
2-20	0.0164 (0.0024, 0.0546)	0.0064 (0.0009, 0.0212)
	Brain/CNS	
5-10	0.0032 (0.0014, 0.0069)	0.0012 (0.0005, 0.0026)
5-20	0.0089 (0.0038, 0.0193)	0.0041 (0.0018, 0.0089)
